# Which risk scenarios can drive the emergence of costly cooperation?

**DOI:** 10.1038/srep19269

**Published:** 2016-01-20

**Authors:** Kristin Hagel, Maria Abou Chakra, Benedikt Bauer, Arne Traulsen

**Affiliations:** 1 Department of Evolutionary Ecology, Max Planck Institute for Evolutionary Biology, D-24306 Plön, Germany; 2 Department of Evolutionary Theory, Max Planck Institute for Evolutionary Biology, D-24306 Plön, Germany

## Abstract

In collective risk dilemmas, cooperation prevents collective loss only when players contribute sufficiently. In these more complex variants of a social dilemma, the form of the risk curve is crucial and can strongly affect the feasibility of a cooperative outcome. The risk typically depends on the sum of all individual contributions. Here, we introduce a general approach to analyze the stabilization of cooperation under any decreasing risk curve and discuss how different risk curves affect cooperative outcomes. We show that the corresponding solutions can be reached by social learning or evolutionary dynamics. Furthermore, we extend our analysis to cases where individuals do not only care about their expected payoff, but also about the associated distribution of payoffs. This approach is an essential step to understand the effects of risk decay on cooperation.

Social dilemmas arise when self-interested individuals have a conflict between their personal gain and the success of their group. Typically, this leads to the ‘free-rider’ problem in the classical public good games^1^,^2^. To overcome such ‘Tragedy of the commons’^2^, different mechanisms have been introduced, such as reward and punishment^3^,^4^,^5^,^6^,^7^,^8^, reputation^9^,^10^,^11^ or repeated interaction between the same players^12^,^13^. These types of measures can promote cooperation, but also affect the degree of cooperation. Individuals can decrease the effective payoff of ‘free-riders’ either directly by punishing them or indirectly by altering their reputation and fostering cooperation by allowing others to select better partners.

Most studies of cooperation within groups have focused on the linear public goods game. A variant, which receives increasing attention, involves reaching a threshold in the number of players — leading to a payoff function that is highly nonlinear^14^,^15^,^16^. The effect of thresholds on cooperation in public good games strongly depends on the type of payoff function implemented^14^. In addition to thresholds, Milinski *et al.*^17^ have introduced a risk probability, such that a group of individuals must cooperate in order to avoid a large loss. These collective-risk social dilemmas have been shown experimentally and theoretically to induce cooperation by the simple inclusion of risk^17^,^18^,^19^,^20^,^21^,^22^,^23^,^24^,^25^,^26^,^27^,^28^,^29^,^30^, and thus may conceptually be considered as an additional mechanism that promotes the evolution of cooperation^31^.

Including risk can help alleviate some uncertainties, such that it may turn the problem of cooperation into a problem of coordination towards a ‘risk free’ state^32^. The degree of risk attributed to the loss can affect the amount contributed by individual group members. In this dilemma, the risk probability can play a major role in the decisions made^17^,^20^,^29^,^30^. In reality, however, this is further complicated since prospects are rarely certain and individuals differ how they perceive the risks involved. So far, the majority of studies has focused on the piecewise step level function, which was the original curve used in experiments^17^. In such a strict setup, individuals are expected to contribute more for high risk probabilities than for low risk probabilities^17^,^18^,^19^,^20^,^21^,^22^. Individuals have a clear cut amount that must be achieved in order to avoid loss. However, what happens if the risks dropped gradually, and at different rates with increasing contributions? Furthermore, how are contributions affected when the risk curve decays gradually rather than abruptly? So far, a thorough theoretical understanding of how different types of risk curves will affect the actions in such a game is still lacking.

The application of various risk curves has broad implications, in particular in the context of climate change^17^,^19^,^20^,^29^,^30^ and can lead to diverse possible cooperative outcomes. These outcomes can depend crucially on how the risk is expected to change. For instance, we could expect to observe behaviours ranging from free-riders to over-compensators, or from fair-sharers to altruists, by manipulating the degree at which the risk decreases with contributions made. Thus, herein we evaluate analytically the relevance of different possible risk curves and show how they influence cooperative behaviours. In particular, we present a general method that can be implemented for any risk function and discuss the outcome for several possible risk curves. We also discuss how strategies will be affected when players have preferences concerning the variation of the payoffs and not simply optimize their expected payoff.

## Model and Analytical Results

We consider a game between *d* players, each one initially receives an endowment *E* and can decide to spend a certain portion *c* of the endowment to reduce a collective risk. Their payoff then depends on their own action and on the action of others: In the collective risk game, the probability of loss *p* depends on the sum of the contributions of all players, *c*_1_ + *c*_2_ + ... + *c*_*d*_, normalized to the total endowment *dE*, where *c*_*j*_ is the contribution of player *j*. With probability *p*, all players lose everything, whereas with probability 1 − *p*, they retain what they have not spent. We call the functions 
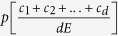
 “risk curves” and assume throughout that the risk can never increase with additional contributions, 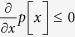. For simplicity, we assume that every individual makes a single contribution, i.e. there are not multiple rounds (in contrast to most experimental approaches to this issue).

Let us first focus on continuous risk curves which start at risk 1 for *c* = 0 and end at risk 0 at *c* = *E*. For 
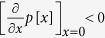
, players can increase their payoffs when they increase their contribution from *c* = 0. Thus, for these risk curves, *c* = 0 is dominated by contributing a very small amount and hence *c* = 0 cannot be a Nash equilibrium. Similarly, *c* = *E* cannot be a Nash equilibrium, as retaining even a very small part of the endowment *ε* is better than keeping nothing, as typically *p*(1 − *ε*) < 1.

We restrict our analysis to a homogeneous population, such that all players have the same strategy characterized by their contribution *c*_*j*_ = *c* for all *j*, i.e. 
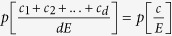
. Players can contribute any amount between 0,..., *E* from their initial endowment *E*, hence the strategy space is continuous. Players retain what they have not contributed from *E* with probability 1 − *p*. Thus, the payoffs follow a binomial distribution. As all players contribute the same amount *c*, the expected payoff 〈*π*〉 is given by


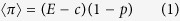


and the variance of the payoff is


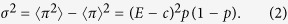


Given two strategies, the stability of fixed points in general multiplayer games can be assessed based on Bernstein polynomials^33^,^34^,^35^. However, we take a more basic approach here, focusing on specific risk curves. It seems clear that the way the risk curve decreases can alter the outcome of the game^14^,^23^. Here, we ask which kind of curves allow for a stable equilibrium with an intermediate level of contributions. One such curve is the stepwise scenario^17^,^21^,^29^, where a player’s decision can change the expected payoff substantially, as it can change the risk for the whole group.

A stable intermediate contribution implies that a player cannot gain from unilaterally deviating from her current strategy. Instead of allowing for all possible deviations, we focus on small strategic changes. Thus, we ask if the payoff of a player increases after slightly decreasing or slightly increasing her current contribution. The payoff of a focal player who invests c^*^ while the remaining *d* − 1 players invest *c* is given by


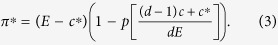


For 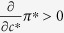, the focal player will increase her contribution. For 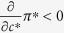, the focal player will decrease her contribution. This is equivalent to the adaptive dynamics approach, which addresses phenotypic evolution in a continuous trait space^36^,^37^,^38^,^39^,^40^. We are interested in a situation where all players invest the same, c^*^ = *c*. At this point, the payoff of the focal player changed with her contribution. This change in the payoff can be expressed as


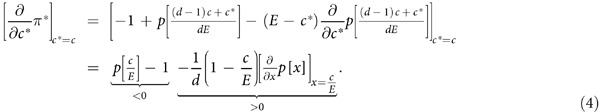


Alternatively, we can express this in terms of the probability *q* = 1-*p* that players retain what they have not spent


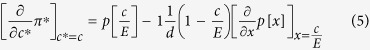


**Figure 1:**
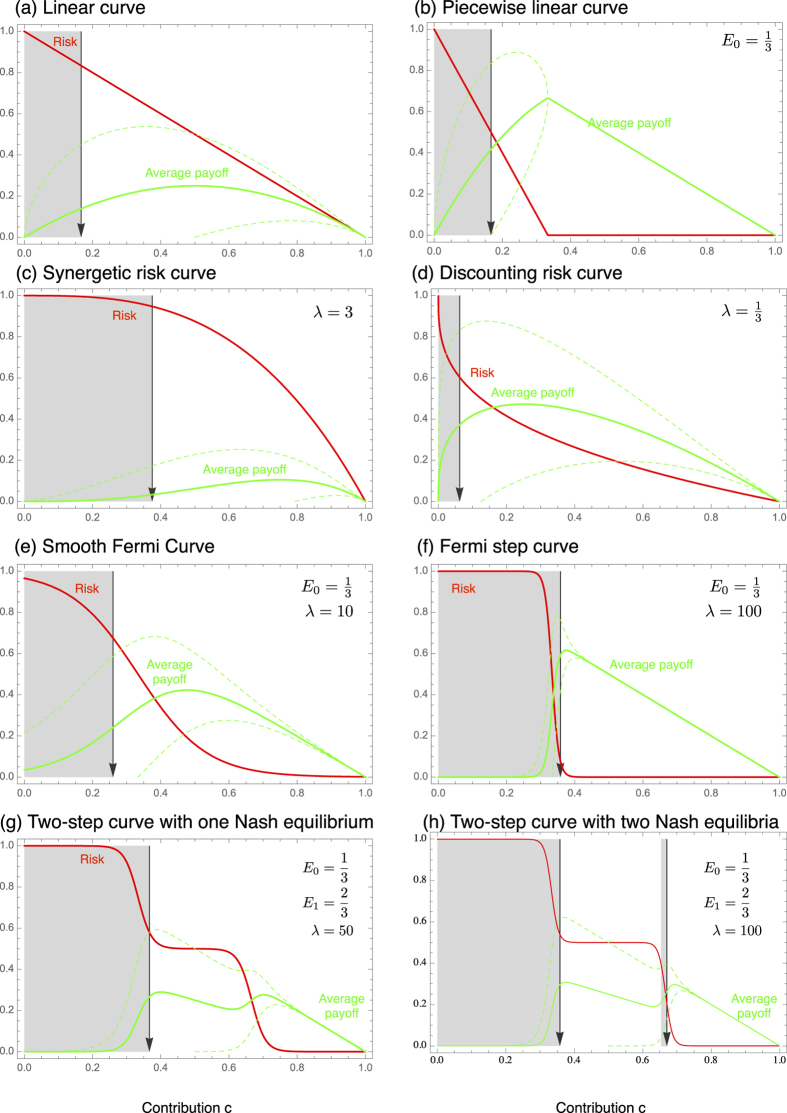
Risk curves, average payoffs and the associated equilibria. Full lines show the risk curve and the average payoffs. Dashed lines depict the standard deviation of the average payoffs calculated from Eq. (2). In grey areas, rational players should increase their contributions, in white areas they should decrease their contributions, which leads to symmetric equilibria depicted by arrows. The curves shown in (**a**–**d**) can be analyzed analytically, see text. The curves (**e**–**h**) have been analyzed numerically. In (**a**–**g**), there is a single symmetric equilibrium, (**h**) depicts an example for a curve with two stable equilibria (in all curves, *E* = 1 and *d* = 5).

A symmetric equilibrium, in which all players contribute the same amount, can be calculated by setting Eq. (4) or (5) equal to 0 and solving for *c*. This leads to an expression for the elasticity of the risk curve,


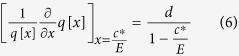


i.e. the relative change of the risk curve necessary for an equilibrium scales linearly with the number of players *d*. As our approach does not allow for arbitrary strategies, e.g. this with a *c* very far from c^*^, it is not precise to call this equilibrium a Nash equilibrium - this needs to be verified in an additional step. For the risk curves discussed in this manuscript, the corresponding extremum is always a maximum and thus stable. This approach can be applied for any particular risk curve. As 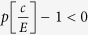, an equilibrium with nonzero contributions can only exist if the risk curve *p*[*x*] decays sufficiently fast. For large *d*, it becomes increasingly difficult to find risk curves which can stabilize cooperation — as in the usual public goods game, larger groups tend to impede cooperation. An extreme case is the step function at a point *E*_0_ (typically used in behavioral experiments with *E*_0_ = *E*/2^17^)


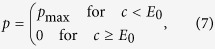


where we have 
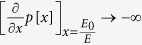
. Thus, there is an equilibrium at *c* = *E*_0_ for any finite number of players *d* if the risk *p*_max_ is sufficiently high, 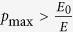. In the following, we discuss several alternative risk curves, which are commonly used or could apply to risky collective games, some are also illustrated in Fig. 1.

### Linear risk curve

The linear risk curve is the simplest decreasing risk function,


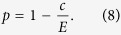


The risk decays from 1 at no contributions to 0 when everyone contributes the whole endowment *E*. As discussed above, players can increase their payoffs when they increase their investment from *c* = 0. They can also increase their payoffs when they decrease their investment from *c* = *E*. The expected payoff for the linear risk curve is given by 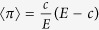, which has a maximum value of 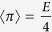 at 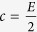. However, this social optimum is not stable, as 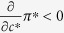 at this point. From Eq. (4), we find instead an equilibrium at


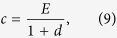


where the expected payoff is 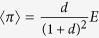. This means that a single player playing this game (*d* = 1) should invest *E*/2 to reach her individual optimum of *E*/4. In a pairwise game, *d* = 2, players should invest a third of their personal endowment. With an increasing number of players, the contributions and the expected payoff decrease with ~*d*^−1^. Consequently, the linear risk curve can only stabilize a minimum level of cooperation in large groups, see Fig. 1a.

We can show that Eq. (9) is actually a Nash equilibrium since a player deviating from such state to any other contribution 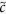 will always have a lower payoff. The payoff of a single deviating player who contributes 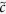 is


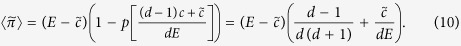


This payoff has a maximum at 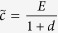, which is exactly the value in Eq. (9). Thus, the equilibrium calculated above — based on small strategy deviations — is also a Nash equilibrium.

We can also modify this curve and set the risk at *c* = 0 to *p*_max_ instead, 
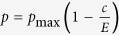
. For sufficiently high *p*_max_, this leads to an equilibrium at 
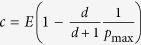
, i.e. at lower contributions for *p*_max_ < 1.

### Piecewise linear risk curve

Alternatively, we can assume that the risk decreases linearly and reaches zero at some value *E*_0_ < *E*, see Fig. 1b. This is described by the risk curve


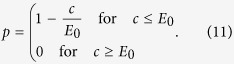


In this case, there can be no equilibrium when *c* > *E*_0_. With smaller *E*_0_, the risk curve decays faster with the contribution *c*. The expected payoff is given by


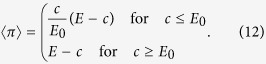


The payoff has a maximum, which reflects the social optimum of the group, at


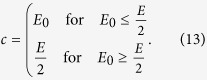


Let us first focus on the case of a fast decaying risk, 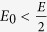. In this case, Eq. (4) leads to an equilibrium at


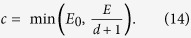


This equilibrium decreases rapidly with increasing *d*.

For slowly decaying risk, 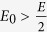, we find an equilibrium at


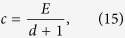


which is always smaller than the social optimum *E*/2 for *d* ≥ 2. This is exactly the result we found for the linear risk curve, which is natural since the expected payoff function in the case of slowly decaying risk is identical to the linear risk curve up to the constant factor *E*/*E*_0_. Thus, the piecewise linear risk curve also leads to a limited level of contributions that decreases rapidly with the number of players.

### Synergy and discounting

In the linear risk curve, every additional contribution decreases the risk in the same way, regardless of the total contributions. We can modify the risk curve such that


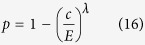


with *λ* > 0. One can think of a synergetic effect of contritbutions, when *λ* > 1 — increasing *c* decreases the risk faster when starting from a higher baseline contribution. Whereas, one can think of a discounting effect when *λ* < 1 — increasing *c* decreases the risk slower when starting from a higher baseline contribution. In both cases, illustrated in Fig. 1c,d, the social optimum is given by


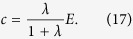


Using Eq. (4), we find an equilibrium at


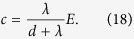


For strong synergetic effects, *λ* ≫ *d*, the equilibrium approaches full contributions, *c* ≈ *E*, but the corresponding average payoff goes to zero, making it difficult to stabilize this high contribution equilibrium. For strong discounting, *λ* ≪ *d*, the equilibrium approaches zero contributions, *c* ≈ 0.

### Fermi function

The step function, Eq. (7), has been used to capture drastic all or nothing events, however, this does not capture intermediate cases. For this, we have to consider a curve that can be used to approximate the step function while also incorporating gradual and smoother change, such as the Fermi function,


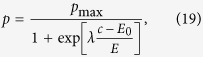


where *p*_max_ is the maximum risk and *λ* is a parameter that controls how fast the risk declines (see Fig. 1e–f), reaching a step function at *E*_0_ in the limit *λ* → ∞. The Nash equilibrium for this curve can only be determined numerically from Eq. (4). For example, with 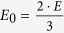, *λ* = 10, *p*_max_ = 1, and *d* = 5, the payoff maximum is at *c* ≈ 0.72, but the symmetric Nash equilibrium is at *c* ≈ 0.44. If we increase *λ* to 20, the payoff maximum is shifted to *c* ≈ 0.64 and the Nash equilibrium to *c* ≈ 0.63 Qualitatively, with a sufficiently high *λ* the risk curve declines fast enough to induce a Nash equilibrium close to *E*_0_ for any number of players *d*.

### More complicated risk curves

Our method extends beyond the few curves we described here. With the same method, we can analyze even complicated risk curves, which do not necessarily have a single stable equilibrium. For instance, if a curve has two areas of fast declining risk, such as


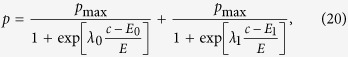


where *p*_max_ is the maximum risk, *E*_0_ and *E*_1_ determine the points of fast declining risk and *λ*_0_ and *λ*_1_ are parameters that control how fast the risk declines. In this case each step can stabilize a separate equilibrium, see Fig. 1g,h. Again the Nash equilibrium for such a curve can only be determined numerically from Eq. (4).

## Evolutionary Dynamics of Strategies

So far, we have calculated the optimal strategies of players given a certain risk scenario. However, it may be problematic to assume that players would immediately use these optimal strategies to play such a game. Thus, we now infer whether it is possible to approach these solutions by a learning process. This can be approached by tools from evolutionary game theory, which have already been used extensively in the discussion of collective risk problems.^17^,^18^,^19^,^20^,^21^,^22^,^23^,^24^,^27^,^28^,^29^,^30^ The basic assumption of evolutionary game theory is that strategies that lead to high payoffs are transmitted within a population, either through a learning process or through evolution. Evolutionary game theory helps to identify particularly interesting strategies, but it can also be used to ask how additional aspects, such as population structure, affect the emergence of such strategies.

Here, we use a simplified version of the simulation approach described in detail in^21^. Briefly, each player has a strategy that is determined by her level of contribution *c*, ranging between 0 and the initial endowment *E*. We sample a group of *d* players from a population of size *N*. Each player contributes an amount of *c*_*j*_ according to her strategy. The contributions of the *d* players are summed up in order to calculate their collective risk of loss of the remaining endowment, *p*, which depends on the risk curve as well as on the total contribution. The payoff of each player is the remaining endowment with probability 1 − *p*, with probability *p* their payoff is 0. In a heterogeneous population, each player’s success depends on the precise composition of her group. Thus, we assume that each player is involved in a large number of games *G* to determine her average payoff given a certain collection of strategies in the population. To obtain stable average payoffs, we assume *G* ≫ *N*.

These average payoffs are then used to model a learning process. We assume that each player switches her strategy to that of a role model, which is chosen proportional to fitness from the whole population. This corresponds to an evolutionary game in a Wright Fisher process^21^,^33^,^34^. In addition, we assume that the new strategy is subject to mutation with probability *μ*, which implies that the new strategy is chosen from a Gaussian distribution of width *ς* around the old strategy.

**Figure 2:**
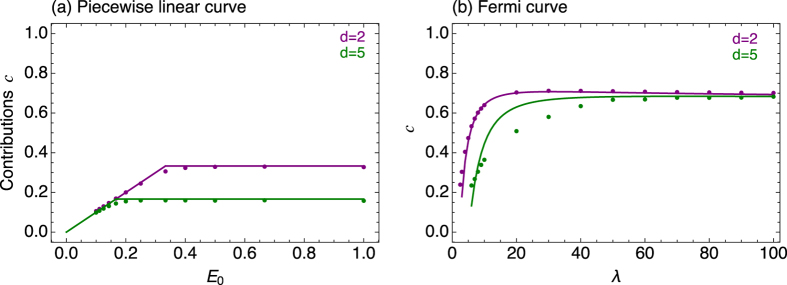
Average contributions in an evolutionary model. Full lines show the Nash equilibrium calculated using Eq. (4). Single points represent the average contribution from ≈2000 realizations (**a**) for the Piecewise linear curve and (**b**) for the Fermi function with 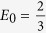. These simulations confirm that the equilibria calculated here can also be obtained as the average of an evolutionary process. For the Fermi curve, the curve must become steeper with increasing *d* to stabilize the equilibrium, as discussed in the main text (*N* = 100, *G* = 1000, *μ* = 0.01, *σ* = 0.01).

Figure 2 show an example that we can recover the strategies discussed above with this simulation approach. However, if the payoff increases only very slowly with increasing contributions, as e.g. in the Fermi curve in Fig. 1, it is very difficult to simulate the emergence of cooperative strategies starting from initially zero contributions.

## Risk Preferences

So far, we have assumed that players optimize their expected payoff and do not take stochastic deviations from this payoff into consideration. However, the vast literature of individual decision making shows that human subjects do not only consider their expected payoff but differ according to risk preferences and prospects, such as being risk averse for gains and risk seeking for losses^35^,^36^,^37^,^38^,^39^,^40^. Risk preferences can also affect cooperative behaviour^41^,^42^,^43^. Thus, we now assume that players consider the distribution of the payoffs in their decisions and explore how this influences overall contributions. For example, a player may be willing to accept a lower expected payoff if it substantially decreases the variance around the expected payoff, i.e. if it reduces the probability of large losses. In the simplest case, this can be modeled by assuming that a player optimizes a utility *u* given by


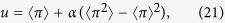


where *α* < 0 describes a risk averse player, *α* = 0 a risk neutral player, and *α* > 0 a risk seeking player. A risk neutral player (as in the examples considered above) ignores variance by only considering the best expected payoff. On the other hand, a risk seeking player chooses a high variance state whereas a risk averse player chooses a small variance state — regardless of a potential decrease in expected payoff.

We follow the same approach as above and analyze the equilibrium of a certain type of risk preferences by substituting the utility function Eq. (19) in Eq. (4). For the linear risk curve, the symmetric equilibria is given by the solution of


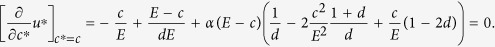


As this expression is cubic in *c*, an analytical solution provides limited insight. We thus focus on numerical techniques to infer the stable points.

**Figure 3:**
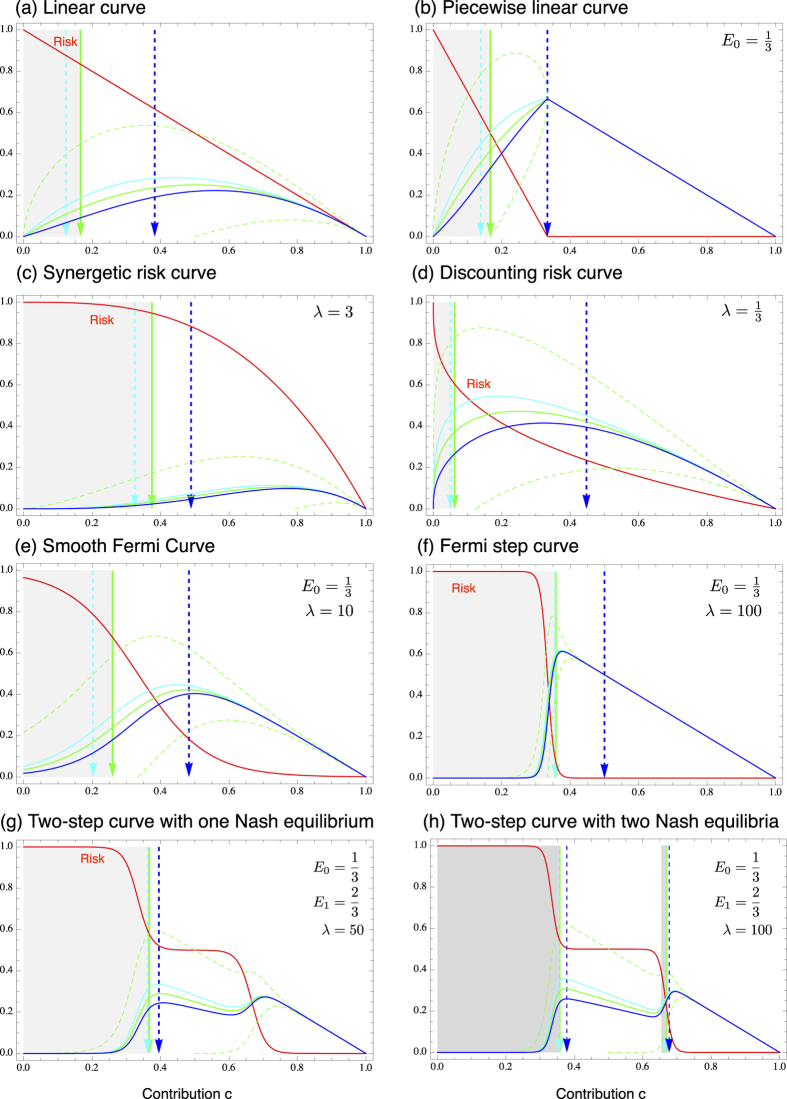
Equilibrium contributions with preferences. In general, risk seeking players (*α* = +0.5 in all panels) tend to contribute less while risk avoiding players (*α* = −0.5 in all panels) tend to contribute more than players who are risk neutral, *α* = 0. The full lines in the colors corresponding to the different risk types are the average payoffs. (**a**) For the linear curve, the asymmetry in the variance leads to a much larger deviation for negative *α*. (**b**) For the piecewise linear curve, the risk avoiding individuals contribute much more and would end up in the social optimum. (**c**–**h**) Depending on the shape of the standard deviations, the change induced by negative *α* can be very small, while the changes induced by positive *α* are much larger (all parameters as in Fig. 1).

Figure 3 shows how the equilibrium change when we introduce preferences. As expected, players with *α* > 0 tend to contribute less, as they can obtain the same utility from lower expected payoff if the variance is larger. For example, in the case of the linear risk curve, 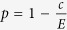 the utility is given by


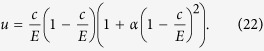


Compared to *α* = 0, positive *α* leads to a social optimum at lower values of *c* and negative *α* leads to a social optimum at higher values of *c*.

We find that players with *α* < 0 tend to contribute more, as they can increase their utility by decreasing their expected payoff and the variance at the same time. Even relatively small changes in the preferences can lead to large deviation from the behavior of risk neutral individuals. It seems worth mentioning that these deviation are typically highly asymmetric: For instance, a risk seeking individual may have essentially the same strategy as a risk neutral individual (with minimal contributions), whereas a risk avoiding individual can have a strategy that leads to substantially larger contributions, cf. Fig. 3.

## Discussion

Risk is an element found in many real life social situations. Typically risk is an exogenous event which we can neither control nor predict. However, in certain situations risk can be influenced by our actions, such as preventing the dangerous consequences of climate change or building dams that defend communities from disastrous flooding. Such scenarios can be modelled and captured using collective risk dilemmas.^17^,^18^,^19^,^20^,^21^,^22^,^23^,^24^,^27^,^28^,^29^,^30^ Individuals must work together to reduce the risk of collective loss. However, depending on the risk, group interest can conflict with personal gain. Thus, cooperation is not only dependent on others but also on the degree of risk.

Here, we show a method that can be used for any risk curve. We consider a simple setting, consisting of homogeneous populations that experience a single risk for all players. Nonetheless, we show curves that look relatively similar can lead to very different outcomes. In general, we can summarise that the equilibrium contribution in such risky situations depends not only on the risk, but also on the first derivative of the risk curve. The most popular scenario in these games considers a step function, which is a special choice from the theoretical perspective, as its derivative diverges at the only point where changes in players’ contributions affect the outcome. Including risk preferences, such that the variance also matters, can substantially affect the outcome: while risk seeker and risk neutral players show similar symmetric equilibria, contributions of risk averse players can differ in all risk curves. This can be attributed to the high variance resulting from the utility functions of risk neutral and risk seeking players. Our theoretical analysis emphasizes that in order to better predict the amount of contributions it is crucial to know exactly how the risk decreases with additional contributions.

Real world social dilemmas are far more complicated due to the heterogeneity between actors^19^,^44^. Additional issues of uncertainty, coordination and communication between the actors are also present in experimental studies that are typically performed over multiple rounds^17^,^20^,^30^,^32^. However, our theoretical study allows the comparison between risk curves that abstracts from these issues and reveals general factors that are crucial in the stabilization of a cooperative equilibrium in these collective risk dilemmas. It turns out that an essential determinant is not the risk curve itself, but the ratio between the derivative of the risk curve and the number of players in the game. As the number of players increases, the curve must become steeper to stabilize a state of intermediate cooperative investments. The step curve, which has been used most extensively in experimental studied, represents a limiting case, as it stabilizes cooperation among any number of players. Furthermore, since the variance vanishes in the special case of the step function with *p*_max_ = 1, utility in symmetric equilibrium coincides with expected payoff independent on risk preferences in that case. In all other cases, it is important to know how much influence a single player’s contribution has on the risk.

In summary, we have shown that collective risk problems are crucially affected by the underlying risk curve and proposed a general method that can be utilised to analyze any risk curve. In a nutshell, for large groups it becomes increasingly difficult to induce cooperation in the face of risk, as the influence of a single player on the collective risk diminishes.

## Additional Information

**How to cite this article**: Hagel, K. *et al.* Which risk scenarios can drive the emergence of costly cooperation? *Sci. Rep.* **6**, 19269; doi: 10.1038/srep19269 (2016).
